# Three-point bending behavior of individual ZnO nanowires studied by *in situ* Laue microdiffraction

**DOI:** 10.1107/S1600576725003668

**Published:** 2025-06-16

**Authors:** Soufiane Saïdi, Michael Texier, Shruti Sharma, Gustavo Ardila, Céline Ternon, Jean-Sébastien Micha, Stéphanie Escoubas, Olivier Thomas, Thomas W. Cornelius

**Affiliations:** a Aix Marseille Univ., Univ. Toulon, CNRS, IM2NP UMR 7334, Marseille, France; b Univ. Grenoble Alpes, CNRS, Grenoble INP, LMGP, 38000 Grenoble, France; c Univ. Grenoble Alpes, Univ. Savoie Mont Blanc, CNRS, Grenoble INP, CROMA, 38000 Grenoble, France; d CRG-IF BM32 Beamline at the European Synchrotron (ESRF), CS40220, 38043 Grenoble Cedex 9, France; e Univ. Grenoble Alpes, CNRS, CEA-IRIG, 38054 Grenoble, France; SLAC National Accelerator Laboratory, Menlo Park, USA

**Keywords:** ZnO, Laue diffraction, nanomechanics, dislocations, nanowires, piezoelectrics, semiconductors, fracture strength, three-point bending, torsion, plasticity

## Abstract

The mechanical behavior of piezoelectric semiconductor ZnO nanowires was studied in three-point bending configuration using the custom-built atomic force microscope SFINX coupled with *in situ* Laue microdiffraction. Besides bending, torsion of the nanowires was shown during mechanical loading. A fracture strength of up to 3 GPa was demonstrated, which is about one order of magnitude higher than that for bulk ZnO. In the case of a piezoelectric material like ZnO, this fracture strength represents the maximum elastic strain that could eventually be converted into electrical energy by the piezoelectric effect. The significantly increased fracture strength found for nanowires compared with bulk ZnO thus offers increased energy-harvesting potential from material flexing. While bulk ZnO is a brittle material, plasticity with the storage of dislocations in the basal plane was shown in the three-point bent ZnO nanowires.

## Abstract 2

The mechanical behavior of piezoelectric ZnO nanowires was studied, demonstrating a fracture strength of up to 3 GPa, significantly higher than that of bulk ZnO. This enhanced strength increases energy-harvesting potential and reveals unexpected plasticity with dislocation storage in the basal plane.

## 1. Introduction

Zinc oxide (ZnO) stands as a cornerstone in contemporary scientific research and technological advancements. Its hexagonal wurtzite crystal structure and its piezoelectric and semiconducting properties have propelled ZnO into various applications (Zhang *et al.*, 2012 ▸; Purica *et al.*, 2000 ▸; Upadhyay *et al.*, 2020 ▸): a wide bandgap of ∼3.37 eV (Mang *et al.*, 1995 ▸) coupled with high exciton binding energy renders ZnO suitable for UV photodetection, LEDs and transparent coatings. On the other hand, both n-type (Thomas, 1959 ▸) and p-type (Joseph *et al.*, 1999 ▸) conductivity enables the fabrication of field-effect transistors, solar cells and sensors. The inherent piezoelectric properties of ZnO allow the conversion of mechanical energy into electrical energy and *vice versa*, playing a vital role in applications such as energy harvesting (Tao *et al.*, 2017 ▸), acoustic wave devices and pressure sensors (Parmar *et al.*, 2019 ▸; Lee *et al.*, 2014 ▸). Additionally, its robust chemical and thermal stability makes it well suited for high-temperature applications, gas sensing (Morisot *et al.*, 2019 ▸) and catalytic processes (King & Nix, 1996 ▸). The exploration of the physical properties of ZnO nanowires (NWs) drives the development of next-generation technologies such as flexible electronics (Núñez *et al.*, 2018 ▸), wearable devices (Sirohi & Chopra, 2000 ▸) and efficient energy-conversion systems (Rensmo *et al.*, 1997 ▸).

The mechanical properties, in particular at the nanoscale, are of prime importance for the application of ZnO nano­structures in future devices. It has been demonstrated that micro- and nano-structures exhibit increased fracture strengths compared with their bulk counterparts, reaching the theoretical limit of the respective material in the case of defect-scarce nanostructures (Wen *et al.*, 2008 ▸; Uchic *et al.*, 2004 ▸; Taylor, 1924 ▸). These ultrahigh fracture strengths allow large elastic deformations, which may eventually be converted into electricity in the case of piezoelectric materials such as ZnO.

In addition, semiconductor materials with deep Peierls valleys, which are brittle at ambient conditions and ductile at elevated temperatures, have been demonstrated to exhibit a brittle-to-ductile transition (BDT) at room temperature for sufficiently small micro- and nano-structures (Hoffmann *et al.*, 2007 ▸). Critical diameters of 300 nm and 1 µm were reported for Si nanopillars (Östlund *et al.*, 2009 ▸; Merabet *et al.*, 2018 ▸) and GaAs micropillars (Michler *et al.*, 2007 ▸), respectively, under which the material undergoes a BDT in compression testing. While impressive elastic deformations of up to 10–15% were reported for ZnO NWs with diameters between 200 and 500 nm in tensile testing, the NWs eventually failed by fracture (Desai & Haque, 2007 ▸). So far, no BDT has been shown either in tension or in bending configuration where both compressive and tensile parts coexist. Such a BDT would be of paramount importance for piezoelectric materials considering that brittle fracture typically sets in at stresses far above the yield stress.

A number of studies exist in the literature on the nanomechanical properties of ZnO NWs (Vlassov *et al.*, 2023 ▸). While most nanomechanical experiments were performed in uniaxial compression or tension, three-point bending, cantilever bending or dynamic resonance experiments have been reported to a lesser extent (Vlassov *et al.*, 2016 ▸; Polyakov *et al.*, 2011 ▸; Fan *et al.*, 2018 ▸). Bending tests are of particular interest regarding energy transducers and energy harvesters where the NWs flex and oscillate as a function of environmental mechanical vibrations. The reported values of Young’s modulus for ZnO NWs range from 15 to 250 GPa where different mechanical testing techniques such as tension, indentation, resonance or three-point bending were employed (Vlassov *et al.*, 2023 ▸). In the case of simulation studies, the reported values range from 210 to 340 GPa (Wang *et al.*, 2016 ▸; Bandura *et al.*, 2017 ▸). This notable dispersion of Young’s modulus is likely attributable to the variety of employed nanomechanical testing techniques and simulations with their respective advantages and limitations.

The large majority of nanomechanical experiments on ZnO NWs were actually performed *ex situ*, whereas *in situ* experiments in combination with scanning or transmission electron microscopy techniques or X-ray diffraction methods offer real-time insights into the mechanical behavior (Cornelius & Thomas, 2018 ▸). In the present work, ZnO NWs were tested *in situ* in three-point bending configuration using the custom-built atomic force microscope (AFM) SFINX in combination with Laue microdiffraction (Ren *et al.*, 2014 ▸, 2020 ▸; Leclere *et al.*, 2015 ▸). This approach gives access to the elastic deformation, as well as to plasticity including the activation of slip systems and storage of geometrically necessary dislocations (GNDs) in the material. The *in situ* nanomechanical tests were complemented by finite-element method (FEM) simulations. In addition, complementary *post mortem* transmission electron microscopy (TEM) confirmed the nucleation and propagation of dislocations in three-point bent ZnO NWs, demonstrating their transition from brittle to ductile behavior.

## 2. Experimental

### 2.1. Samples

**Figure 1:**
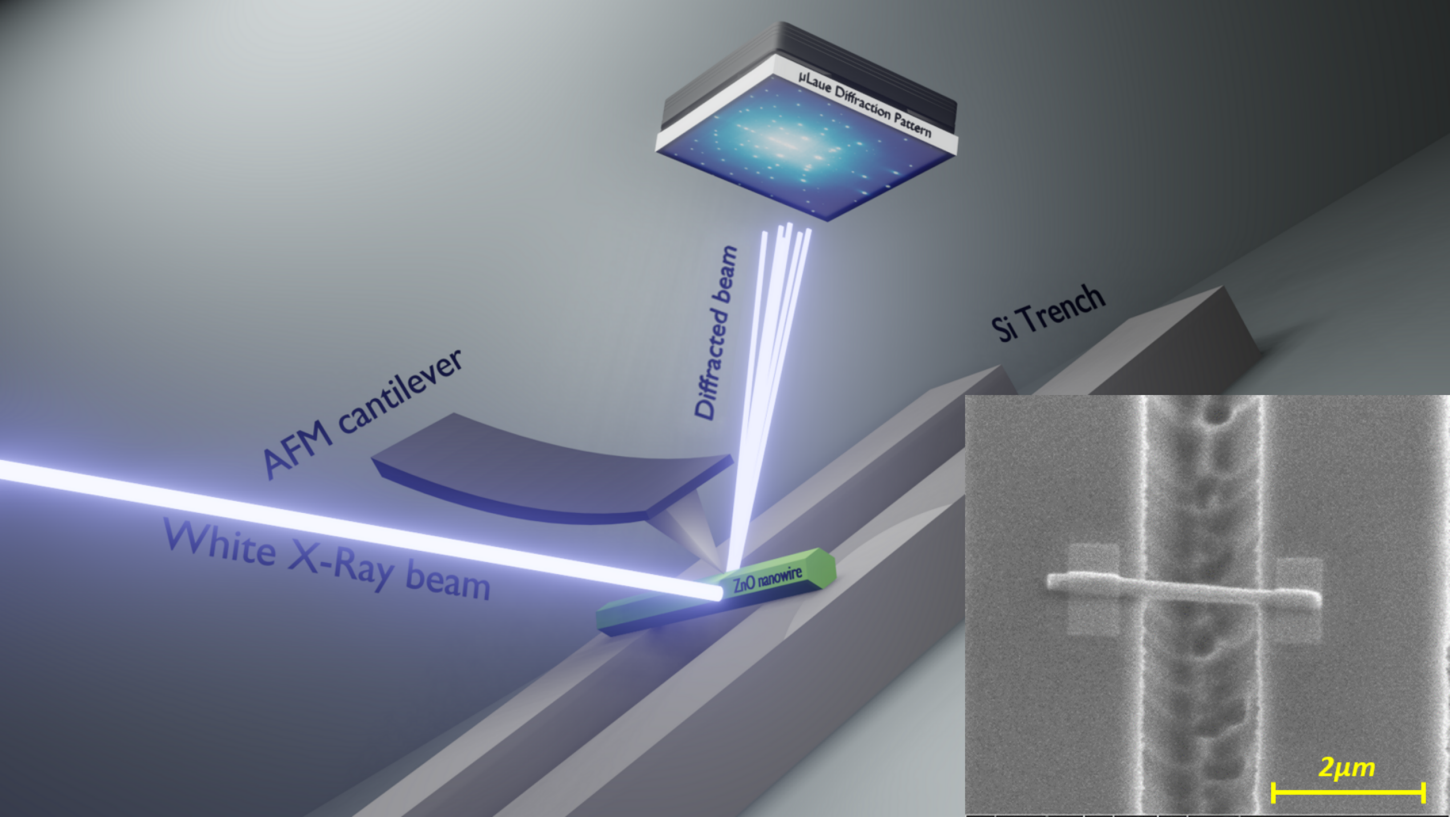
Schematic of the experimental setup (not to scale), showing a suspended ZnO NW and a corresponding experimentally measured Laue microdiffraction pattern. The inset shows a SEM image of a suspended ZnO NW crossing a Si micro-trench and being fixed at both ends by electron-beam-induced deposition of Pt.

High-quality ZnO NWs with a diameter of ∼200 nm and a length of ∼4 µm were fabricated by a hydrothermal process. A ZnO seed layer was first deposited on a Si(100) substrate using a sol–gel procedure that enables one to selectively tune the mean grain size, surface coverage rate and texture coefficient of the ZnO films. Then, ZnO NWs were grown on the ZnO films by hydrothermal synthesis at ambient pressure using an optimized protocol. More details on the fabrication can be found elsewhere (Demes *et al.*, 2016*a* ▸,*b* ▸). A scanning electron micrograph of a dense forest of 200 nm-diameter ZnO NWs is presented in Fig. 1.1 of the supporting information. The ZnO NWs were detached from the growth substrate by ultrasonication, which also lead to their fracture into few-micrometre-long sections. The solvent containing ZnO NWs was then drop-casted onto a Si substrate patterned lithographically with 2 µm wide and 1 µm deep micro-trenches. Some of the NWs crossed these micro-trenches forming suspended nanobridges. These suspended NWs were thoroughly attached at both ends by electron-beam-induced deposition of Pt in a focused ion beam (FIB) microscope (see inset of Fig. 1 ▸). In this work, three ZnO NWs were studied, which are called from hereon NW I, NW II and NW III. The NW parameters of length, suspended length, diameter and misorientation angle of the NW with respect to a perpendicular crossing of a trench are summarized in Table 1 ▸.

### 2.2. Laue microdiffraction

Laue microdiffraction was performed at the French CRG-IF beamline BM32 at ESRF in Grenoble (France). The incident polychromatic X-ray beam with an energy range of 5–25 keV was focused down to 300 (H) × 500 (V) nm on the sample surface, which was inclined by 40° with respect to the incident beam, using a pair of Kirkpatrick–Baez mirrors. The diffracted X-rays were recorded using an sCMOS detector (Photonic Science) with 2018 × 2016 pixels and a pixel size of 73.4 µm, installed at a distance of 77 mm from the sample position at an angle of 90° with respect to the incident X-ray beam (Ulrich *et al.*, 2011 ▸). The Laue microdiffraction patterns were indexed by means of the *LaueTools* software giving access to the UB orientation matrix of the respective crystal (Micha & Robach, 2013 ▸). The fluorescence yield was monitored using a Röntec XFlash 1001 energy-resolved point detector.

For *in situ* three-point bending experiments, the custom-built *in situ* AFM SFINX, equipped with a self-sensing and self-actuating Akiyama probe, was installed on the *xyz*-translation stage of the Laue microdiffraction setup. A schematic drawing of the experimental setup is presented in Fig. 1 ▸ showing an exemplary scanning electron micrograph of a suspended ZnO NW and an experimental Laue microdiffraction pattern. The SFINX Si tip and the focused polychromatic X-ray beam were aligned with respect to a selected suspended ZnO NW by atomic force topography imaging in tapping mode and mapping of the 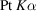 and 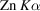 fluorescence yields, respectively. The SFINX Si tip was positioned ∼2 µm above the center of the suspended ZnO NW, and the external excitation of the Akiyama probe was switched off to avoid any fatigue-like mechanical testing during the three-point bending experiments. The ZnO NW was then loaded mechanically by displacing the piezoelectric stage holding the Akiyama probe with a constant speed (ranging from 12 to 25 nm s^−1^). After reaching a pre-defined displacement, the Akiyama probe was retracted with the same speed. Laue microdiffraction patterns were recorded during the complete loading–unloading cycle with a frequency of 0.2 Hz, corresponding to a displacement of 60–125 nm of the piezoelectric stage.

### 2.3. TEM

*Post mortem* TEM was performed on three-point bent NWs for further analysis of their microstructure using a Cs-corrected transmission electron microscope (FEI TITAN 80-300) operated at 300 kV. High-resolution (HR)-TEM images were taken using a negative spherical-aberration coefficient, both to improve spatial resolution and to reduce delocalization effects (Texier & Thibault-Pénisson, 2012 ▸). The NWs were harvested from the patterned Si wafer and electron-transparent lamella for TEM analysis were prepared by FIB micromachining. For this purpose, a platinum protective shield was first deposited onto the NW by electron-beam-induced deposition in a FIB microscope (FEI Helios 600 NanoLab). Using an acceleration voltage of 30 kV, a slice was then cut parallel to the axis of the NW to withdraw it together with the supporting Si substrate (see Fig. 1.3 in the supporting information). Thinning of the lamella was performed using decreasing voltages and ionic currents down to 1 kV and 17 pA, respectively, to minimize the sample damaging. Finally, the lamella was thinned down to a thickness of less than 20 nm by 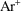 ion milling using a Fischione Nanomill device operating at a 500 V accelerating voltage.

### 2.4. FEM simulations

The NW deformation was additionally simulated within continuum elasticity by the FEM using COMSOL *Multiphysics* (https://www.comsol.com/), taking into account the NW geometry, the anisotropic elasticity constants of ZnO NWs and the fact that the ZnO NW is thoroughly clamped at both ends. Table 2 ▸ summarizes literature values of the elastic constants of bulk ZnO (Morkoç & Özgür, 2008 ▸) and ZnO NWs (Huang *et al.*, 2009 ▸), demonstrating a large variety in these parameters. Given that the experiments performed in the present work involve single-crystal ZnO NWs, particular attention was directed towards a seminal investigation conducted by Huang *et al.* (2009 ▸) that focused on studying ZnO NWs by *in situ* TEM using a mechanical resonance method.

## 3. Results

### 3.1. Force calibration

**Figure 2:**
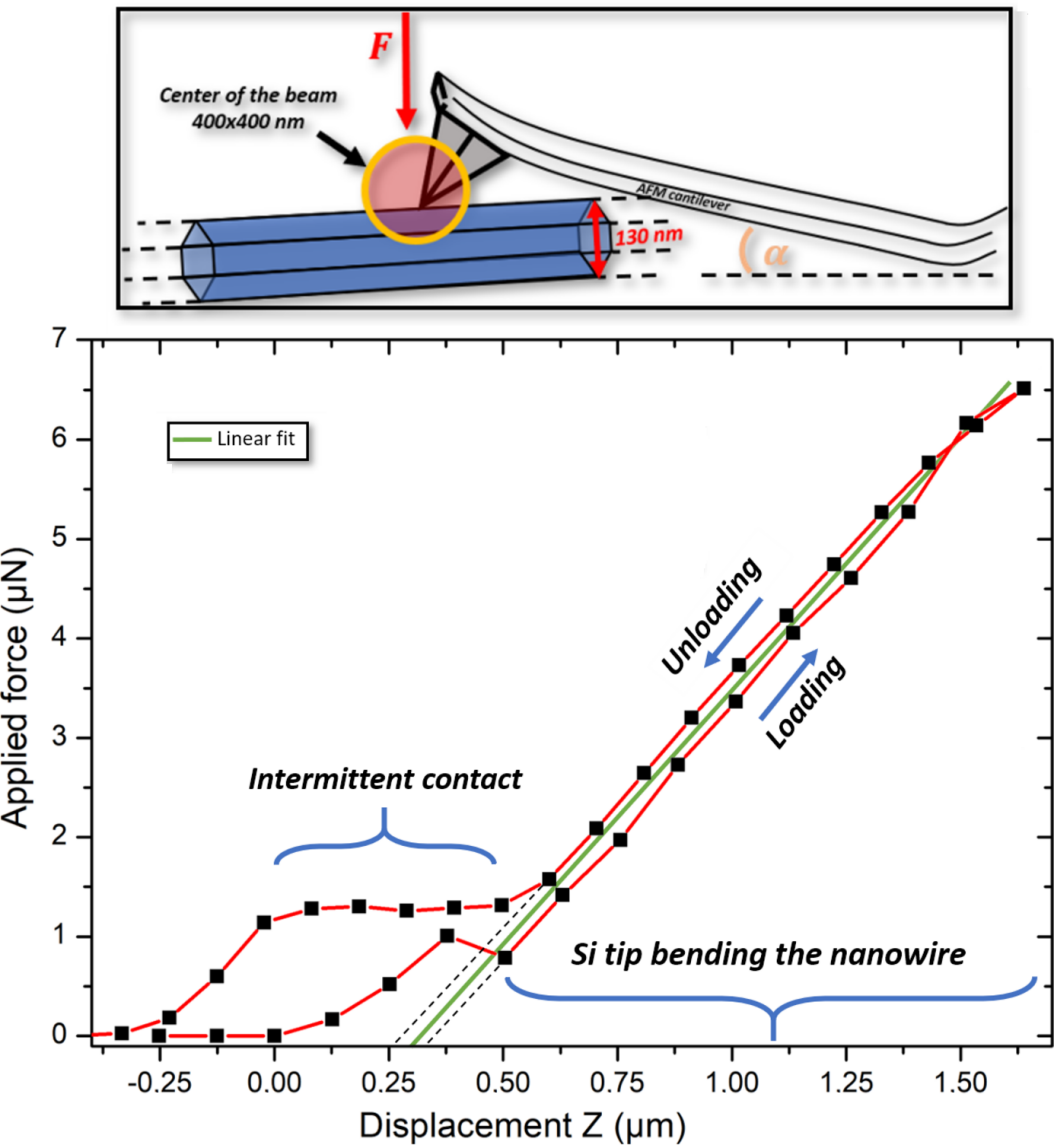
Applied force as a function of the displacement of the piezoelectric stage carrying the Si cantilever inferred from the rotation angle of the Si lattice planes of the SFINX tip (illustrated in the schematic above).

The custom-built AFM SFINX lacks the capacity for direct measurement of the applied force due to the absence of either an optical laser feedback like in ordinary AFMs or a deflection-sensitive cantilever. In order to circumvent this shortcoming, the deflection of the Si cantilever of the Akiyama probe was determined directly by Laue microdiffraction (Lauraux *et al.*, 2021 ▸). Therefore, the incident polychromatic X-ray beam was focused on the Si tip during the three-point bending of a ZnO NW staying within the elastic regime of the nanostructure (see inset of Fig. 2 ▸). The displacement of the Si Laue spots on the detector was monitored, and the rotation angle α of the Si lattice planes of the SFINX tip (which coincides with the rotation of the lattice planes of the cantilever as a whole) during the mechanical loading was inferred from the UB orientation matrices obtained by the indexing of the Si Laue microdiffraction patterns. Considering the length of the cantilever as *L* = 300 nm and a cantilever stiffness of *k* = 5 N m^−1^ (as provided by NanoAndMore GmbH), the applied force *F* was inferred from the rotation angle of the Si tip lattice planes as described by Lauraux *et al.* (2021 ▸): 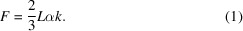
Due to the fact that the piezoelectric stage is actually open-loop, the real speed of the loading and unloading varies by ∼4%. This discrepancy was taken into account, resulting in a loading–unloading curve that overlaps almost perfectly [in contrast to Lauraux *et al.* (2021 ▸)], as presented in Fig. 2 ▸. The interaction of the SFINX tip with the NW is characterized by an intermediate contact regime during the first 500 nm of movement of the piezoelectric stage after the first interaction between NW and tip. This intermediate contact regime may include sliding of the AFM tip on the NW surface. Once the strong physical contact is established, the applied force increases linearly with the displacement *z* of the piezoelectric stage. During unloading, the intermediate contact range is prolongated by ∼250 nm due to the adhesion of the SFINX tip to the NW before the tip completely detaches from the nanostructure. The linear part of the loading–unloading curve with a slope of 5 µN µm^−1^ (which agrees very well with the stiffness value provided by the manufacturer) is used as calibration for the applied force for all following three-point bending experiments on NWs with similar diameters (considering similar NW stiffness) where only the displacement of the piezoelectric stage after the first contact of the SFINX tip with the studied NW is known. Considering both the intermediate contact regime and the intersection of the extrapolated linear part of the loading–unloading curve with the *x* axis leads to an uncertainty of the exact point where the Si tip is in hard contact with the NW. This uncertainty results in an error of 0.74 µN in the absolute value of the applied force, while the relative variations of the force can be measured with a very high accuracy of 114 nN.

### 3.2. Three-point bending

**Figure 3:**
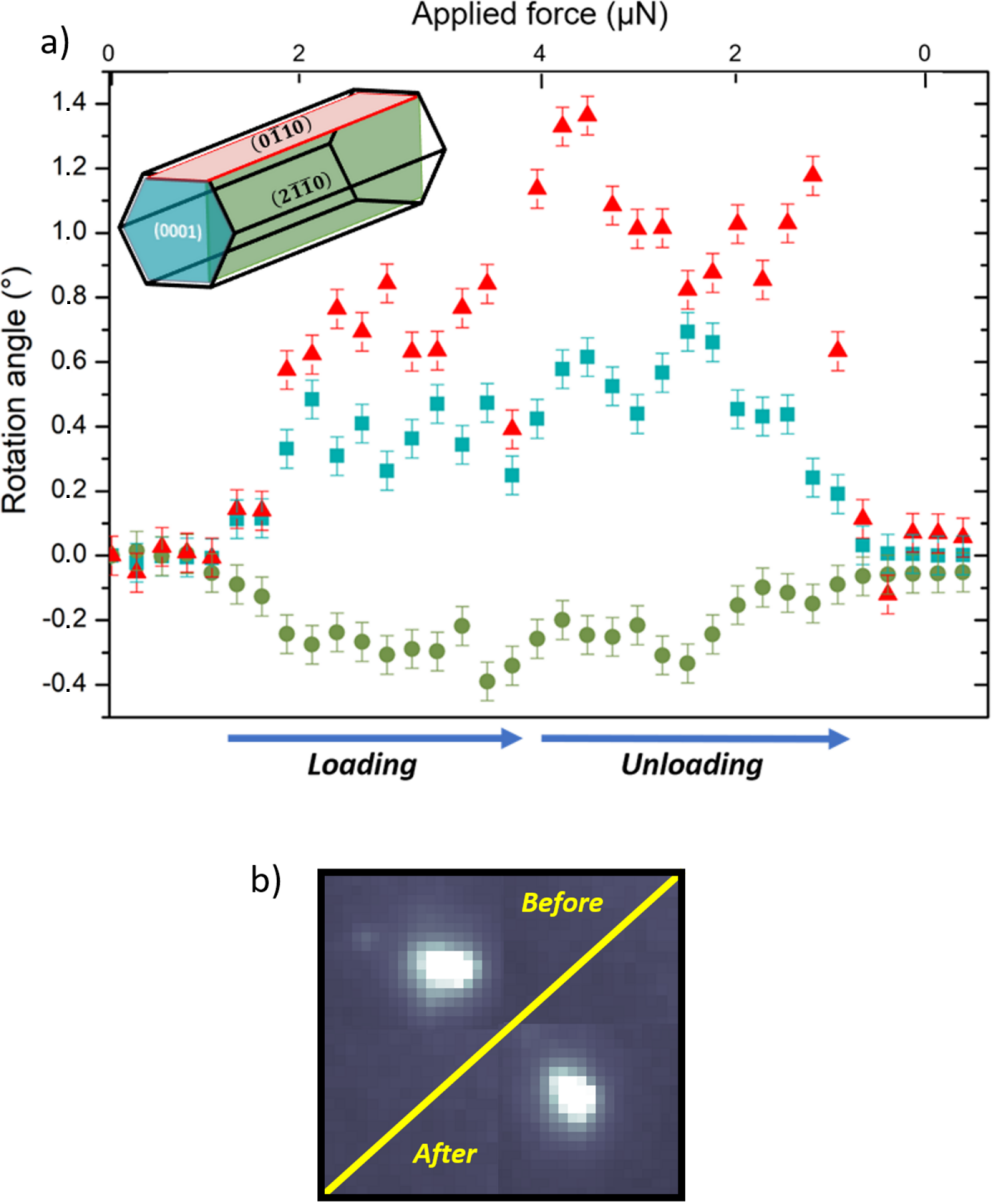
(*a*) Rotation angles of the three orthogonal lattice planes (0002) (squares), 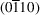 (triangles) and 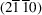 (circles) in ZnO NW I during three-point bending. The inset shows the crystalline orientation of the NW with its growth direction along [0001] lying flat on one of its 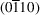 side facets. (*b*) The ZnO 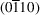 Laue spot before and after the three-point bending test, demonstrating that no plasticity was actually induced within the probed volume.

Prior to the *in situ* three-point bending tests, the NW orientation was determined by Laue microdiffraction, revealing that the NW growth is along the *c* axis and all NWs lie on one of their 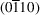 side facets. To determine the deformation of ZnO NW I during three-point bending tests, the rotation angles of the three orthogonal lattice planes (0002), 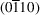 and 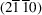 were inferred from the Laue microdiffraction patterns recorded *in situ* during the mechanical loading–unloading cycle [Fig. 3 ▸(*a*)]. While for a perfect three-point bending test only the orientation of the (0002) and 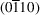 lattice planes is affected, an additional rotation of the 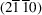 lattice planes is detected, indicating the presence of an NW torsion. This torsion is ∼4 times smaller than the NW bending itself. The fact that the rotation angles for all three lattice planes return to their initial values within the experimental error bars after complete unloading indicates a fully elastic behavior. This is supported by the shape of the Laue diffraction spots, which is the same before and after mechanical loading [as demonstrated for the ZnO 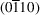 Laue spot in Fig. 3 ▸(*b*)], thus confirming that no defects were introduced and stored in the material in the probed volume.

**Figure 4:**
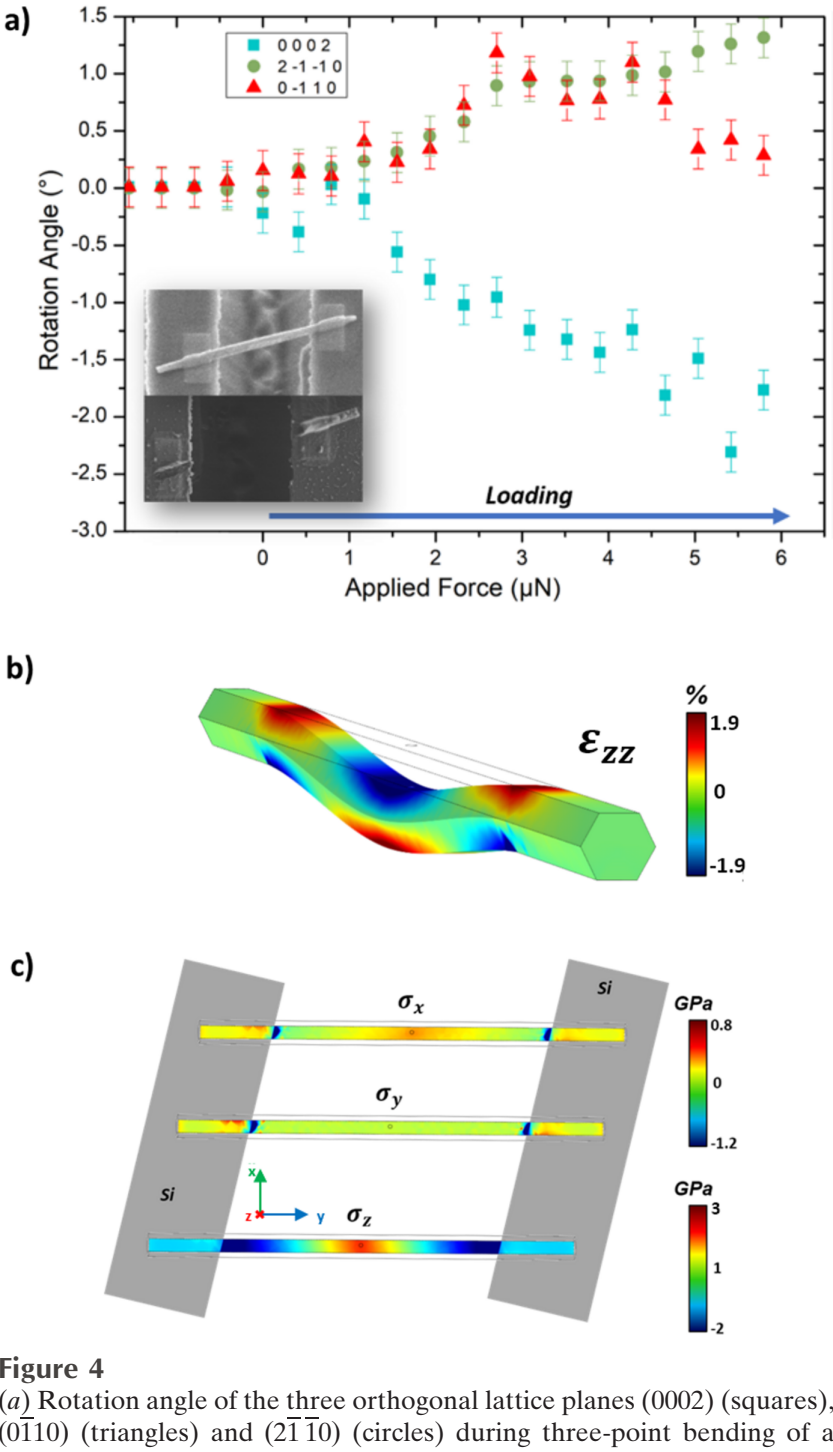
(*a*) Rotation angle of the three orthogonal lattice planes (0002) (squares), 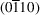 (triangles) and 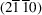 (circles) during three-point bending of a ZnO NW with a diameter of 200 nm and a suspended length of 2.5 µm until failure. The inset shows SEM images before and after mechanical testing, confirming NW fracture. (*b*) Strain (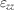) and (*c*) stress (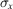, 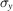 and 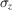) (of the lower NW facet) in the ZnO NW at the highest applied load of 5.79 µN, calculated by FEM simulations considering the same loading and positioning configuration as in the experiment. The *z* direction shows a high stress of 3 GPa at the loading point, while the *x* and *y* directions are clearly affected by the positioning of the NW along the trenches, with the stress near the embedded parts highest in the opposite parts of each region.

The fracture strength was determined by a three-point bending test of ZnO NW II until failure occurred. As in the previous case, the three orthogonal lattice planes (0002), 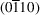 and 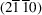 rotate during mechanical loading, indicating bending as well as NW torsion [Fig. 4 ▸(*a*)]. Here, ZnO NW II was loaded up to a force of 6.8 µN (±0.74 µN) before NW fracture occurred, as confirmed by *post mortem* scanning electron microscopy (SEM) imaging [see inset of Fig. 4 ▸(*a*)]. While the rotation angles of the 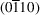 and 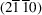 lattice planes follow each other very closely, they actually deviate from each other for *F* > 4.5 µN. This deviation might originate either from the slipping of the SFINX tip on the NW facet or from plasticity in the NW before the actual fracture. Figs. 4 ▸(*b*) and 4 ▸(*c*) present the stress 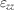 and the strain (of the lower NW facet), respectively, along *x*, *y* and *z* at the highest applied load just before fracture, which were simulated by the FEM. Here, the NW growth axis is oriented along the *y* direction and the loading direction along *z*. For calculations, a circular contact area with a diameter of 15 nm corresponding to the radius of curvature of the SFINX tip was considered for applying the mechanical load. The maximum bending of the NW just before fracture amounts to 70 nm. The stress and strain are concentrated at the loading point in the center of the suspended NW and close to the two clamping points. The strain 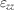 ranges from −1.9 to +1.9%. The stresses 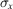 and 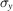 in the NW’s lower facet are found to vary between −1.2 and +0.8 GPa, while 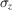 ranges from −2 to +3 GPa. The largest negative stresses for all three components are found close to the clamping positions, while the maximum stress is located at the loading position. Inhomogeneous strain distributions in the NW on top of the Si supports close to their edges are visible, which are induced by the non-orthogonal crossing of the micro-trench by the NW.

**Figure 5:**
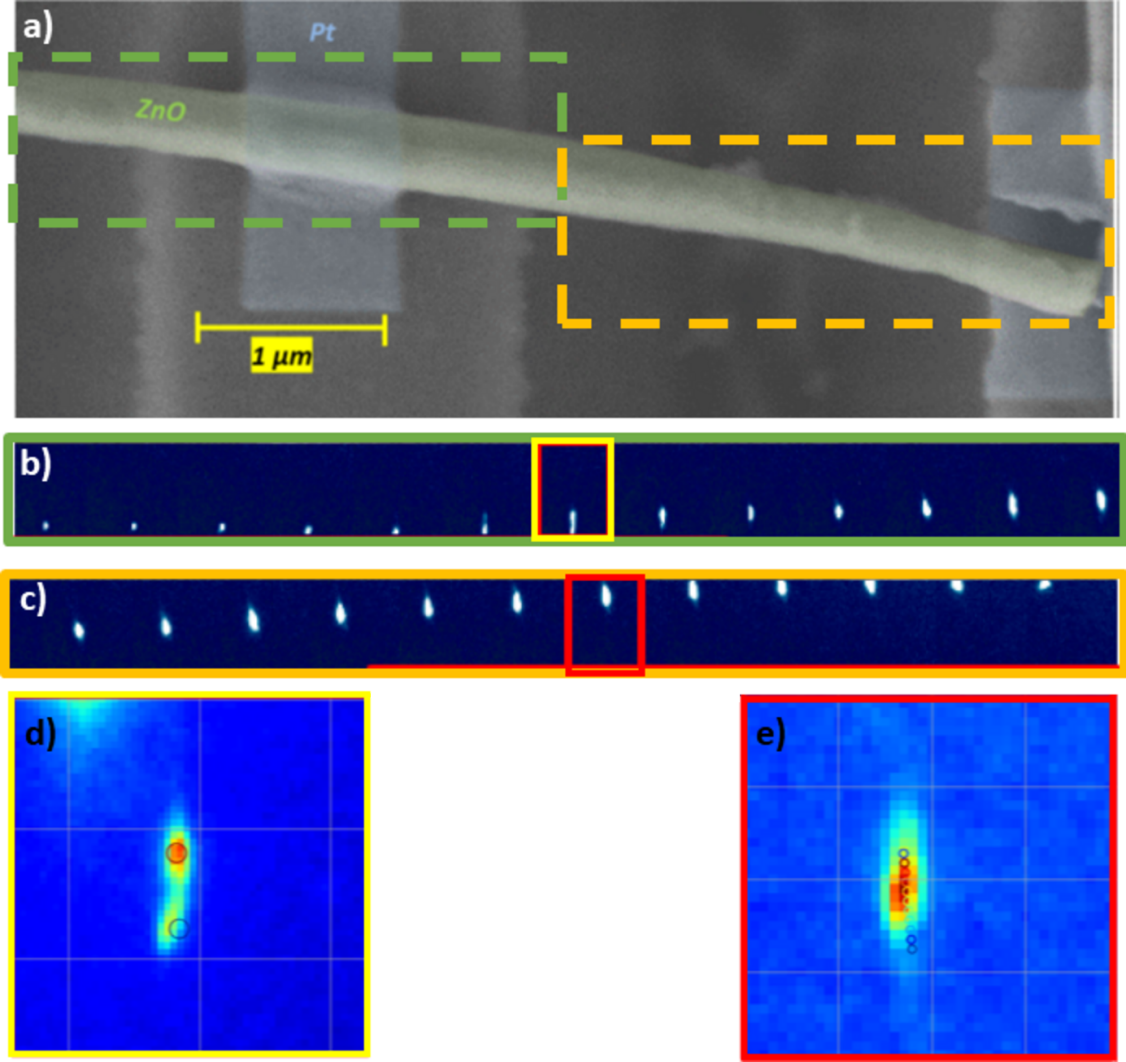
(*a*) *Post mortem* scanning electron micrograph of ZnO NW III after three-point bending testing, demonstrating irreversible deformation. Sequence of the [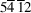] Laue spot measured along (*b*) the left-hand part of the NW and (*c*) the suspended NW part after nanomechanical testing. Zoom-in of the [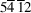] Laue spot measured (*d*) in the embedded part of the NW and (*e*) in the suspended NW part, showing a splitting and a streaking of the diffraction peak, respectively. The circles indicate simulated Laue spots considering a rotation of the NW around the [0001] axis. In the case of the split Laue spot, a rotation of 0.20° reproduces the splitting well.

To further elucidate potential plasticity in ZnO at the nanoscale, ZnO NW III was mechanically loaded and unloaded with a maximum applied stress of 0.5 GPa at the loading point and a strain of 0.15% both in the center and close to the clamping points. It shows the same behavior as the previous NWs with bending as well as torsion. The rotation angles for the three orthogonal lattice planes were found to return to their initial values, indicating a fully elastic behavior (see Fig. 1.2 of the supporting information). However, in this case, the shape of the Laue diffraction spot of the completely unloaded ZnO NW III changed in comparison with its initial shape, implying the storage of defects and thus the activation of plasticity. The irreversible deformation of the NW is confirmed by the *post mortem* SEM image presented in Fig. 5 ▸, showing a clear bending of the right part of the NW. Laue microdiffraction patterns were recorded all along the NW after nanomechanical testing. The [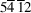] Laue spot is exemplarily displayed below the *post mortem* SEM image. The Laue spot moves on the detector as a function of the position along the NW, revealing a variation of the crystalline orientation. In addition, a splitting of the Laue spot is evident in the clamped part on the left-hand side of the NW, while a pronounced streaking of the spot is visible in the suspended NW. The splitting and streaking of the Laue spot are both oriented in a similar direction, indicating that the crystal lattice rotates almost around the same axis at these two positions. The rotation axis corresponds to the [0001] direction (as illustrated by the simulated displacement of the Laue spot, indicated by black circles), signifying an NW torsion. The splitting of the Laue spot in the clamped area is well reproduced by a local rotation 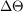 of the crystal by 0.20° around the [0001] axis. The Laue spot splitting is a clear indication of sub-grain formation, which can be related with the aggregation of GNDs in a geometrically necessary boundary. Considering Nye’s law, the number of stored GNDs *n* can be inferred from this local rotation of the crystalline lattice: 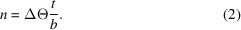
Taking into account the NW thickness *t* of 260 nm and assuming dislocations in the basal plane with the Burgers vector of magnitude *b* = 0.324 nm, the number *n* of stored GNDs within the probed volume is ∼3.

**Figure 6:**
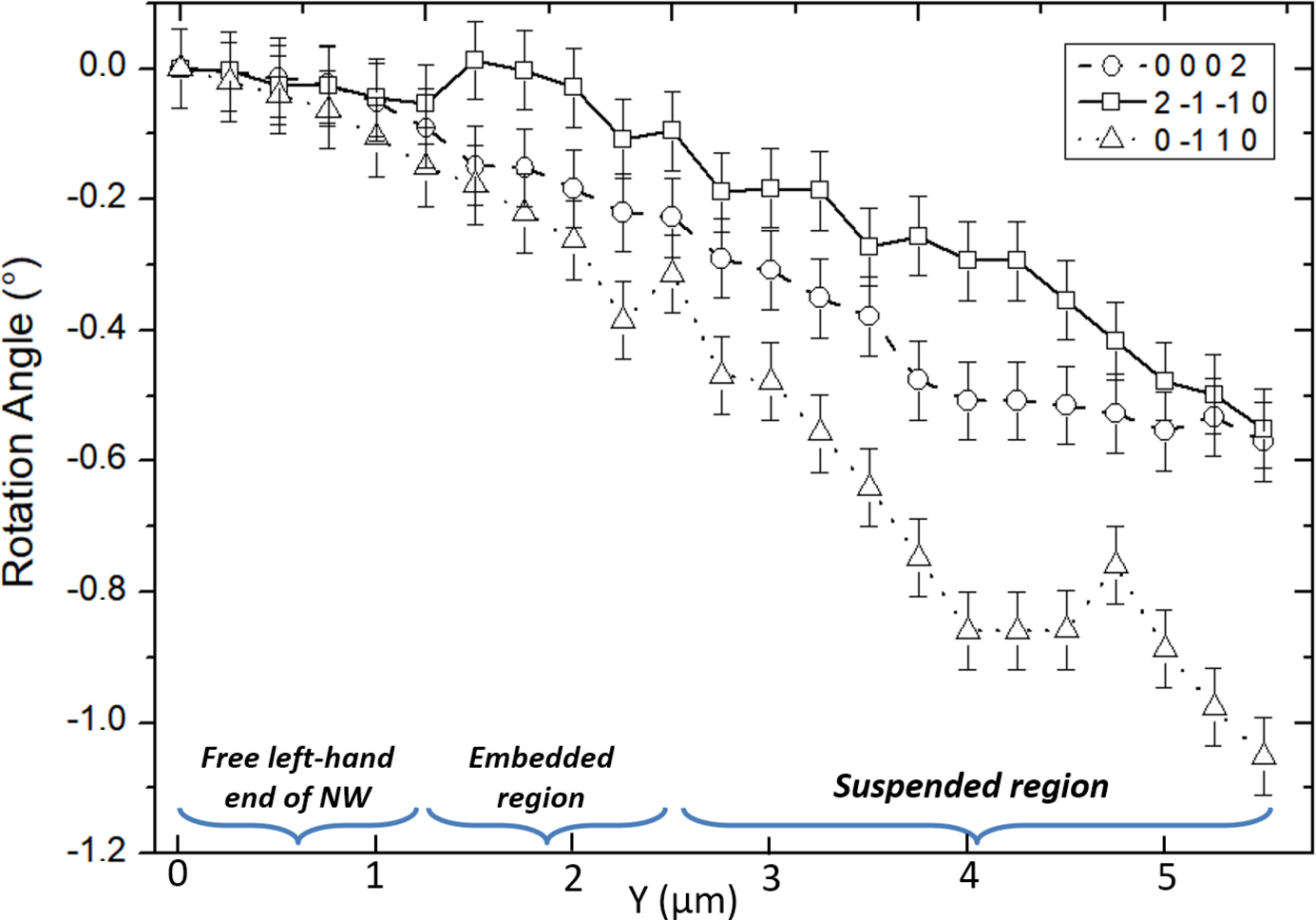
Rotation angle of the three orthogonal lattice planes (0002) (squares), 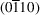 (triangles) and 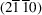 (circles) measured along the length of ZnO NW III in steps of 200 nm after complete unloading.

The simulation of an NW torsion does not perfectly reproduce the streaking of the second highlighted Laue spot in Fig. 5 ▸. In addition, the streaking direction of the Laue spot varies along the NW and, as mentioned above, the Laue spot moves on the detector as a function of the position along the NW. The irreversible NW deformation is thus more complex than a simple torsion around the *c* axis. From the displacement of the Laue spots on the detector as a function of the position along the NW, the rotation of the three orthogonal lattice planes (0002), 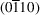 and 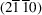 was inferred (Fig. 6 ▸), demonstrating both bending and torsion of the NW. This irreversible rotation of the NW crystal shows plastic deformation and the creation of defects in the ZnO NW.

### 3.3. Microstructural study

To further investigate the irreversible deformation of ZnO NW III and the nature of the defects involved in the above-mentioned plastic deformation, the microstructure of this NW was analyzed by *post mortem* TEM. For this purpose, an electron-transparent lamella containing a [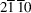]-oriented slice was extracted from the NW using FIB micromachining and then thinned down by 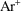 ion milling. Due to the pronounced bending of the NW at one of its extremities, this part was progressively eroded and disappeared during the thinning process of the TEM lamella.

**Figure 7:**
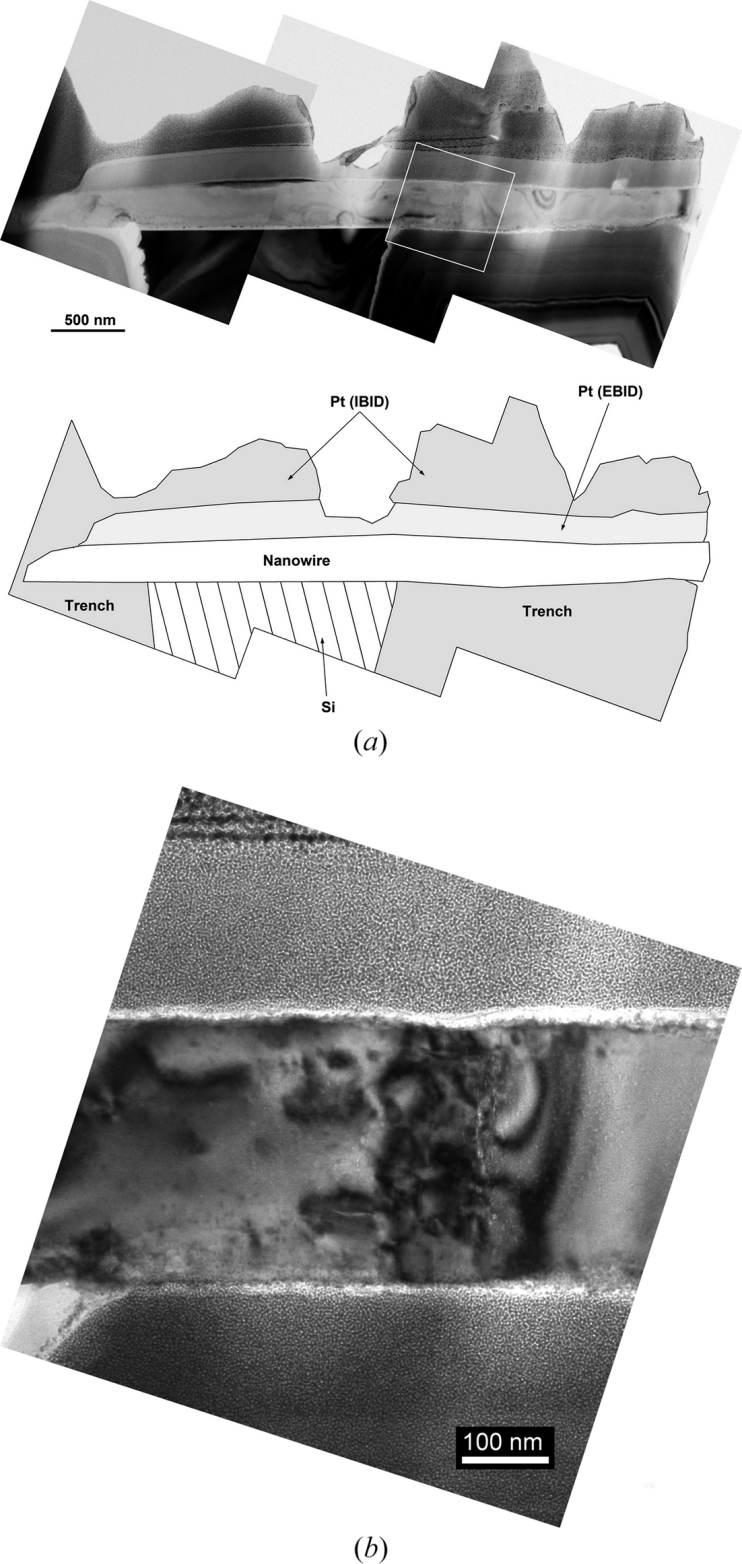
(*a*) Assembly of bright field (BF) TEM images of the plastically deformed ZnO NW, observed in cross section, with accompanying schematic showing the key parts. (*b*) Higher-magnification BF image of the area marked by a square in the top image. This zone, located at the edge of the trench, contains numerous extended defects.

Observation of the NW cross section by conventional TEM imaging (*cf.* Fig. 7 ▸) allowed us to draw a general picture of the deformation: the main part of the NW is free of defects while the observed extended defects are concentrated in a very narrow region in the vicinity of one edge of the Si support. This is in agreement with the FEM simulations predicting high stress concentrations in this region. Circular bent contours just below the surface in the central part of the NW are notably visible, probably resulting from an unintentional nanoindentation by the SFINX tip. Surprisingly, no defects were observed either within the NW below this zone or further towards the bent end of the NW.

**Figure 8:**
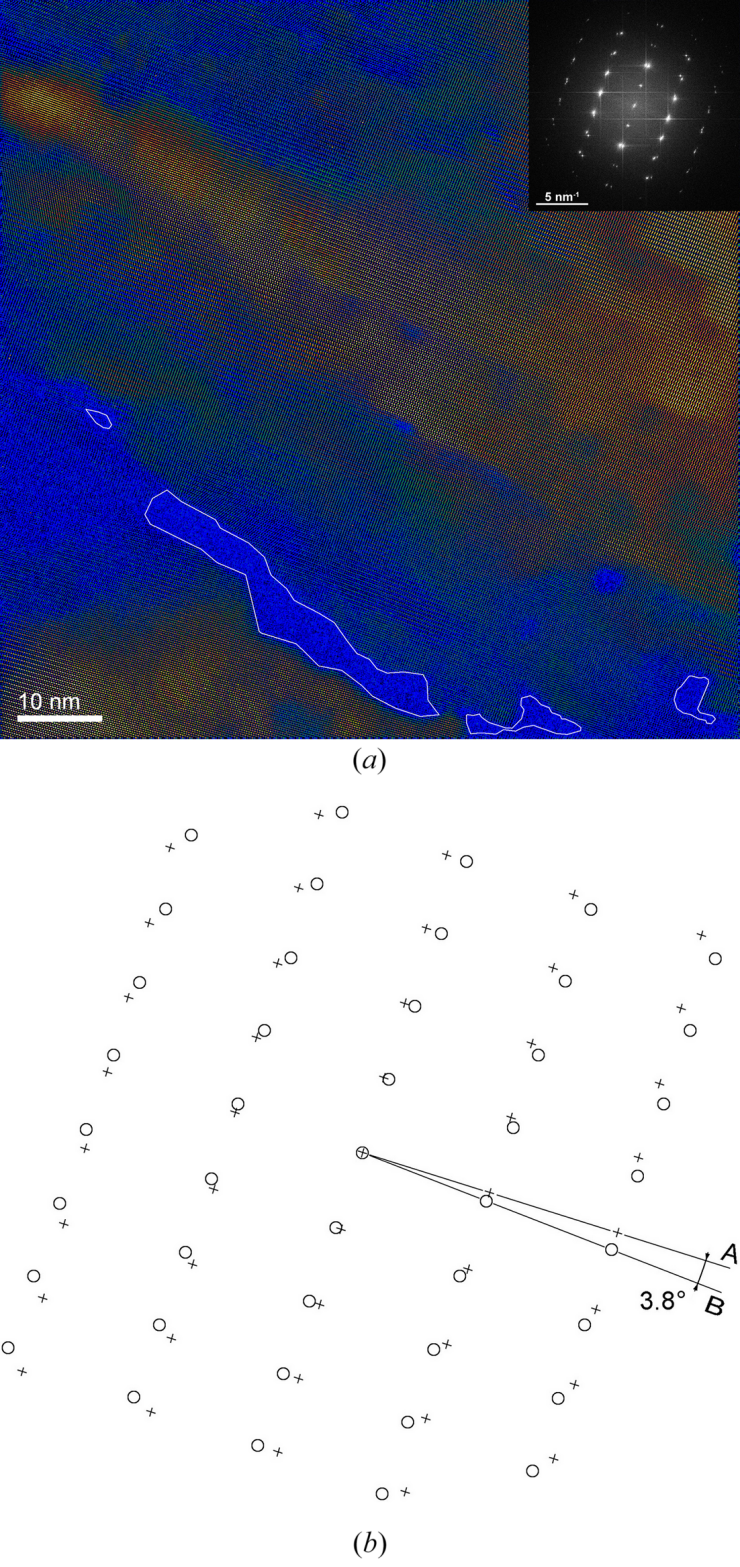
(*a*) HR-TEM image filtered using the average background subtraction filtering method. The intensity is shown in colorscale. Two differently oriented crystalline parts of the NW are separated by a disordered transition zone containing microcracks (see highlighted areas). (*b*) Schematic drawing of the spots observed in the FFT of the image [inset in (*a*)]. The two crystalline parts are parallel to the [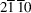] crystallographic axis and rotated by an angle of 3.8° with respect to each other.

HR-TEM imaging was performed on an NW part located close to the edge of the trench corresponding to the framed zone in Fig. 7 ▸. This HR-TEM imaging, presented in Fig. 8 ▸, shows two differently oriented portions of the NW in the vicinity of a few microcracks and extended defects. Both NW portions are observed parallel to the [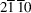] crystallographic axis, as shown by the fast Fourier transform (FFT) of the image. The analysis of the FFT indicates that the two parts are rotated with respect to each other by an angle of 3.8° around the [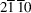] direction (*i.e.* perpendicular to both the NW axis and the SFINX tip displacement direction). The area imaged by TEM is much smaller than that probed by Laue microdiffraction, resulting in the different amount of rotation observed by these two techniques.

**Figure 9:**
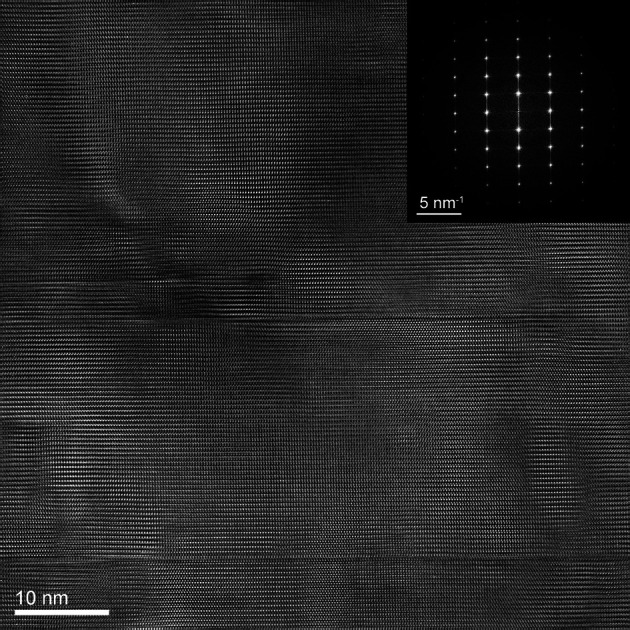
Average background subtraction filtered HR-TEM image and corresponding FFT of the area located at the corner of the trench. The observed extended defects appear as straight lines of variable length parallel to the [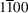] direction and correspond to dislocations extended in the (0001) planes.

**Figure 10:**
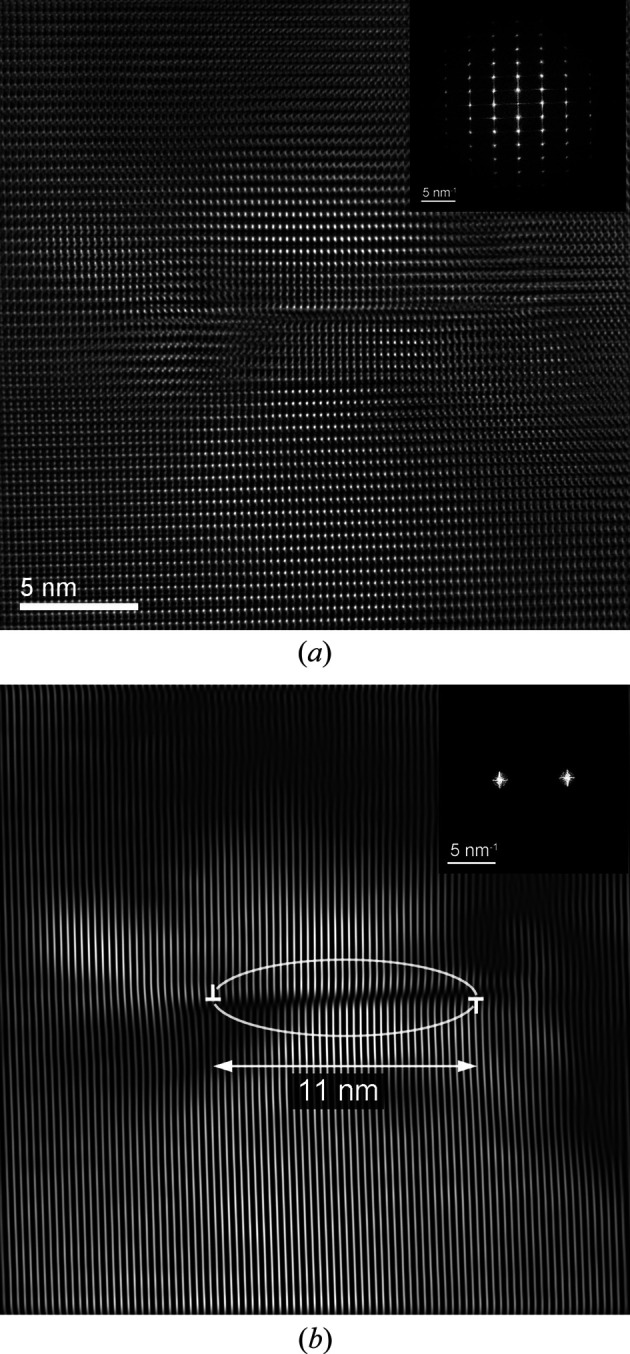
(*a*) Average background subtraction filtered HR-TEM image and corresponding FFT of a region containing an isolated dislocation loop. (*b*) Image filtered using the 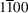 symmetrical reflections. A scheme showing the approximate positions of the two opposing 60° segments of the dislocation is superimposed on the image.

In this region close to the corner of the Si support, numerous defects extending in (0001) planes are also observed (see Fig. 9 ▸). As the presence of stacking faults has not been detected in the examined areas, these defects likely consist of perfect *a*/3〈
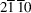
〉 dislocation loops. Depending on the local thickness of the observed zone, they appear as straight lines of variable length, parallel to the [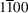] direction. A defect with a short extension is exemplarily shown in Fig. 10 ▸. Filtering of the FFT of the HR-TEM image shows that this defect consists of a small dislocation loop that is partially or completely contained within the TEM lamella.

**Figure 11:**
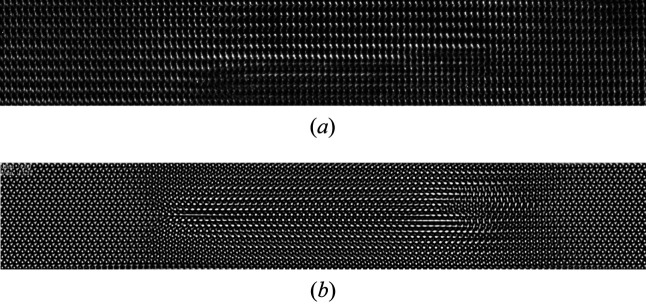
(*a*) Detail of an HR-TEM image showing two small dislocation loops in close basal planes. The local thickness of the sample, derived from image simulation of a defect-free region of the sample close to the observed region (see Fig. 1.5 in the supporting information), is estimated to be 17.2 nm. (*b*) Multislice simulation showing the local contrast variations produced by two dislocation loops with Burgers vectors **b** = 1/3[11
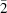
0] in a similar configuration to that observed in the experimental image (*t*: 17.2 nm; C1: −12 nm; C3: −7 µm). The simulation does not take into account the slight misorientation of the sample with respect to the 〈11
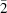
0〉 crystallographic axis as seen in the experimental image.

Although a very high defect density is observed in the studied region, the experimental HR-TEM images do not allow for a direct identification of the dislocations (*e.g.* Burgers vector, core structure) due to the continuous contrast variations in the basal planes containing the observed defects. An example of such a characteristic contrast is shown in Fig. 11 ▸, where the observed defective region is likely to contain two very close dislocation loops in parallel basal planes. It can therefore be assumed that the observed dislocations consist of small dislocation loops with a diameter not exceeding the thickness of the TEM lamella in the investigated regions (between 10 and 50 nm).

## 4. Discussion

All ZnO NWs tested in the three-point bending configuration in this work demonstrated a more or less significant torsion during loading. These findings can be explained either by a misalignment of the SFINX tip with respect to the center of the NW side facet or by the orientation of the NW with respect to the Si micro-trenches. As demonstrated by FEM simulations of ZnO NW II, the fact that the NW does not cross the micro-trench at a right angle induces inhomogeneous strain distributions in the NW, which eventually may induce a torque that leads to an NW torsion around its *c* axis. The HR-TEM analysis also showed that we have a preferred direction of dislocation growth in the length of the basal plane, and the screw segments are perpendicular to the NW axis, which favors a torsion of the NW.

Fracture strengths of up to 3 GPa and maximum strain of ∼1.9% were demonstrated for three-point bent ZnO NWs. These values are about one order of magnitude larger than those in bulk ZnO and are in line with other studies reporting fracture strengths of 2–8 GPa and maximum strains of a few percent for ZnO NWs (Wen *et al.*, 2008 ▸; Hoffmann *et al.*, 2007 ▸; Roy *et al.*, 2017 ▸).

In the case of the irreversibly deformed ZnO NW III, split Laue spots as well as streaked Laue spots were shown. The splitting is a clear indication of the misorientation of two parts of the crystal within the probed volume, which agrees with a geometrically necessary boundary formed by GNDs at this position. The TEM microstructural analysis of the region close to the left-hand Si support revealed the presence of a high density of small basal dislocation loops, while local rotations of the basal planes were detected in the FFT of the HR-TEM images. The dislocation configurations locally responsible for either a torsion or a bending of a crystal can be considered schematically as acting either as twist or as tilt sub-grain boundaries (SGBs), respectively. In the former case, a pure twist SGB is formed by screw dislocation segments extending in the plane perpendicular to the axis of rotation, whereas in the latter case, a pure tilt boundary is formed by an array of edge dislocation segments with Burgers vectors perpendicular to the axis of rotation (Basu & Barsoum, 2007 ▸). Between these two extremes, the presence of mixed SGBs produces both macroscopic torsion and bending of the crystal. Assuming that only basal dislocation slip has been activated in the studied NW, the two observed local mechanical responses (torsion around the [0001] axis or bending leading to the rotation of the NW planes) can result from the activation of the three types of perfect dislocations in the basal planes, depending on their respective proportions of screw and edge components.

The macroscopic deformation of the NW shown by both the Laue spot streaking and the *post mortem* SEM images may result from the propagation of extended defects, some of which are stored in the NW after unloading leading to permanent deformation. However, no dislocations were found by TEM, either below the loading position or in the right-hand part of the NW, while another large part of the NW was lost during lamella preparation.

## 5. Conclusions

In conclusion, the mechanical behavior of ZnO NWs with a diameter of 200 nm has been investigated by three-point bending tests in combination with Laue microdiffraction using the custom-built atomic force microscope SFINX. Besides a vertical bending of the NWs, torsion has been shown that may originate either from a misalignment of the SFINX tip with respect to the center of the NW side facet or from the NW orientation with respect to the Si micro-trench. Despite the comparatively small angle of torsion, *in situ* Laue microdiffraction allows for detecting it and accurately measuring the actual nanomechanical deformation with a high precision. The *in situ* testing of different NWs has further shown both plastic deformation and brittle fracture. Fracture strength of up to 3 GPa has been demonstrated, which is one order of magnitude larger than for bulk ZnO. Such elevated fracture strength offers the possibility to deform the NWs to much larger strain that may eventually be converted into electrical energy by the piezoelectric effect, and thus significantly increase the energy conversion efficiencies compared with bulk materials. Moreover, while bulk ZnO is a brittle material, ductile behavior is shown for the first time in three-point bent NWs with the nucleation and storage of dislocations in the basal plane.

## Supplementary Material

Supporting information. DOI: 10.1107/S1600576725003668/te5150sup1.pdf

## Figures and Tables

**Table 1:** The ZnO NWs studied in this work and their parameters

NW number	Length (µm)	Suspended length (µm)	Diameter (nm)	NW angle (°)
I	3.8	1.8	213	3.7
II	5.27	2.5	195	18
III	6.2	2	265	5

**Table 2:** Elastic constants 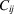 (GPa) of ZnO

Source/parameters	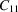	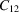	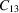	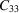	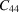	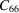
Carlotti *et al.* (1995 ▸)	206	117	118	211	44.3	44.6
Carlotti *et al.* (1987 ▸)	157	89	83	208	38	34
Bateman (1962 ▸)	209.7	121.1	105.1	210.9	42.47	44.29
Ahuja *et al.* (1998 ▸)	230	82	64	270	75	62
Huang *et al.* (2009 ▸)	202.9	132.4	113.0	213.6	34.19	35.24

## Data Availability

The data that support the findings of this study are available on reasonable request from the corresponding author.
