# Supplementary material for: Three-point bending behavior of individual ZnO nanowires studied by in situ Laue microdiffraction
Source: J Appl Crystallogr. 2025 Jun 16;58(Pt 4):1149–58. doi: 10.1107/S1600576725003668 (PMC12321028; doi:10.1107/S1600576725003668)
Supplement: Supplementary file 1 [file j-58-01149-sup1.pdf]

JOURNAL OF APPLIED CRYSTALLOGRAPHY

Volume 58 (2025)

Supporting information for article:

Three-point bending behavior of individual ZnO nanowires studied by *in situ* Laue microdiffraction

Soufiane Saidi, Michael Texier, Shruti Sharma, Gustavo Ardila, Céline Ternon, Jean-Sébastien Micha, Stéphanie Escoubas, Olivier Thomas and Thomas Cornelius

1 Supplementary material

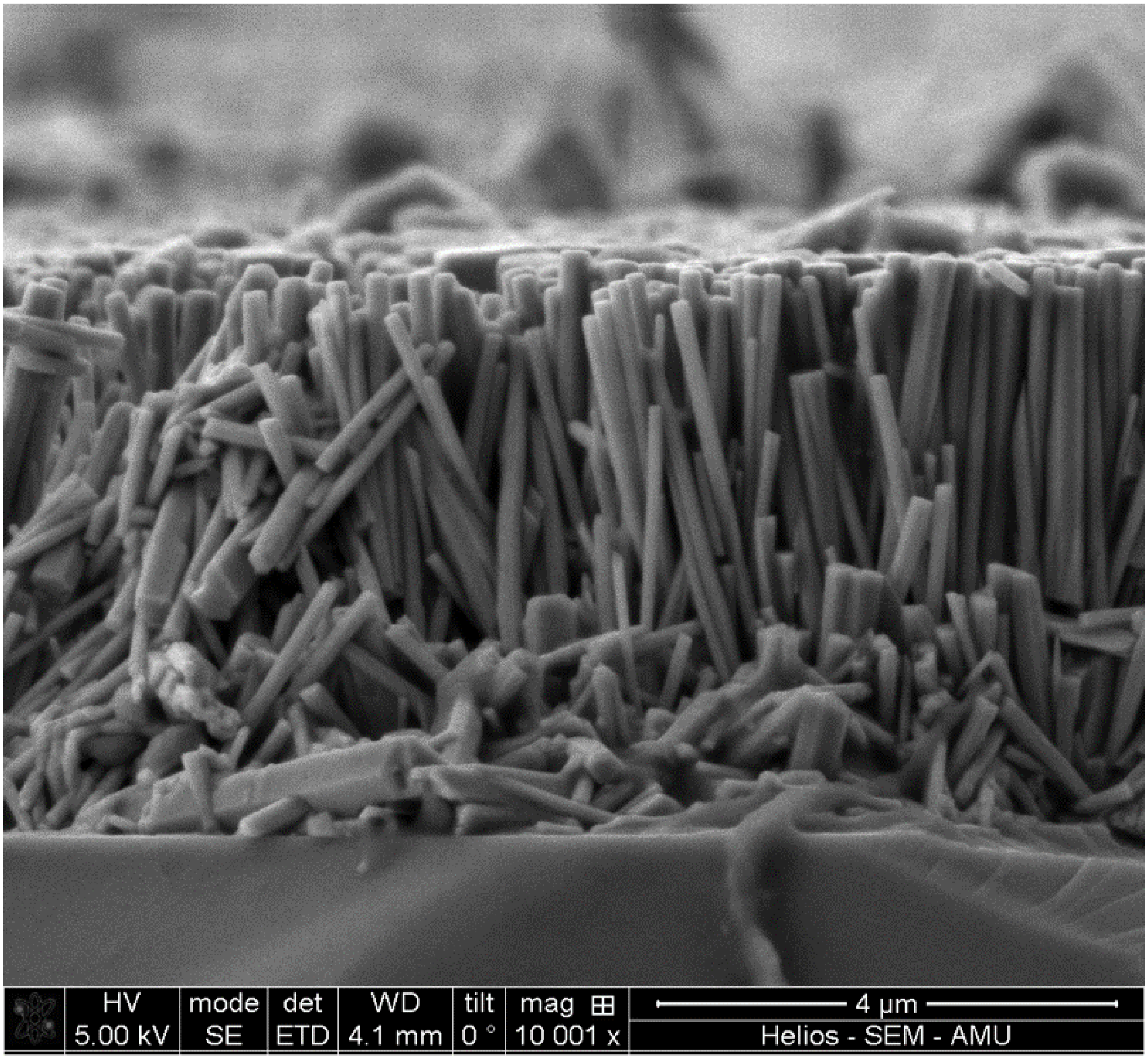

Figure 1.1: Scanning electron microscopy image of the cross-section of a sample containing a dense forest of ZnO NWs on top of a Si substrate.

Scanning electron microscopy image of a dense forest of ZnO nanowires on a Si substrate grown by liquid phase synthesis using the hydrothermal method. The as-grown nanowires measure between 200 and 300 nm in diameter and are 1.5 to 4 μm long.

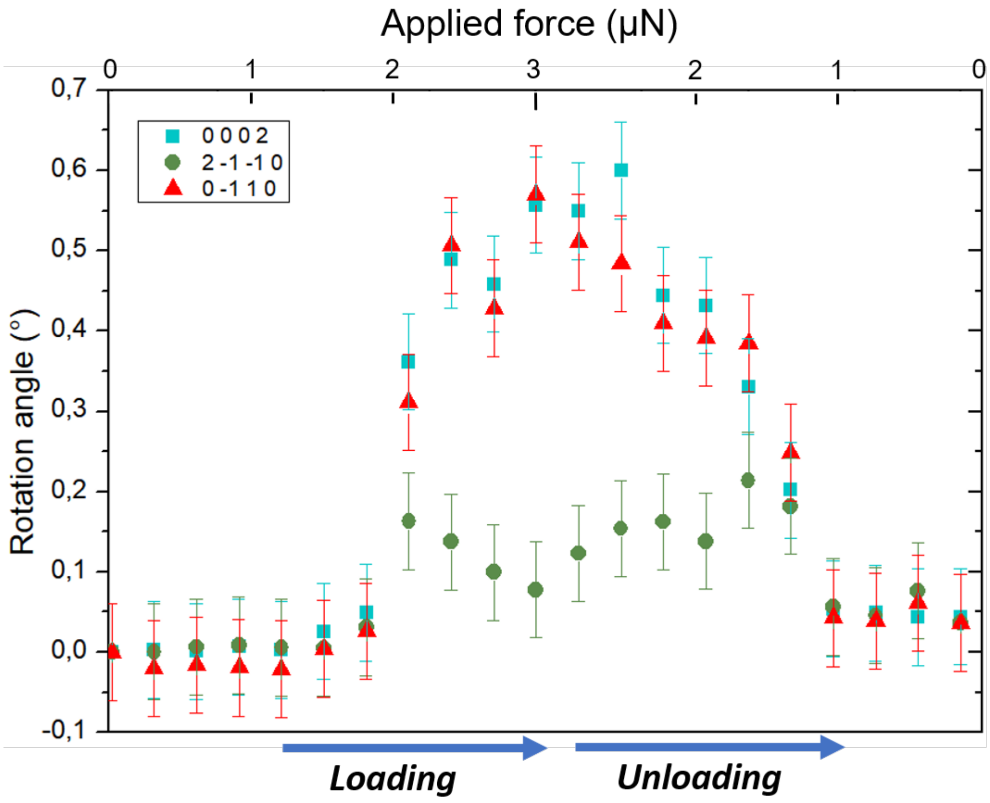

Figure 1.2: Rotation angle of the three orthogonal lattice planes (0002) (□), (0$\bar{1}$10) (△), and (2$\bar{1}\,\bar{1}$0) (◯), during three-point bending of ZnO NW III

The rotation angle of the three orthogonal lattice planes (0002), (0$\bar{1}$10), and (2$\bar{1}\,\bar{1}$0) for the ZnO NW III during three-point bending is presented in Fig. 1.2. As for all other tested ZnO NWs all three lattice panes rotate during loading indicating both bending and torsion. All three lattice planes return to their initial value after complete unloading within the experimental uncertainty.

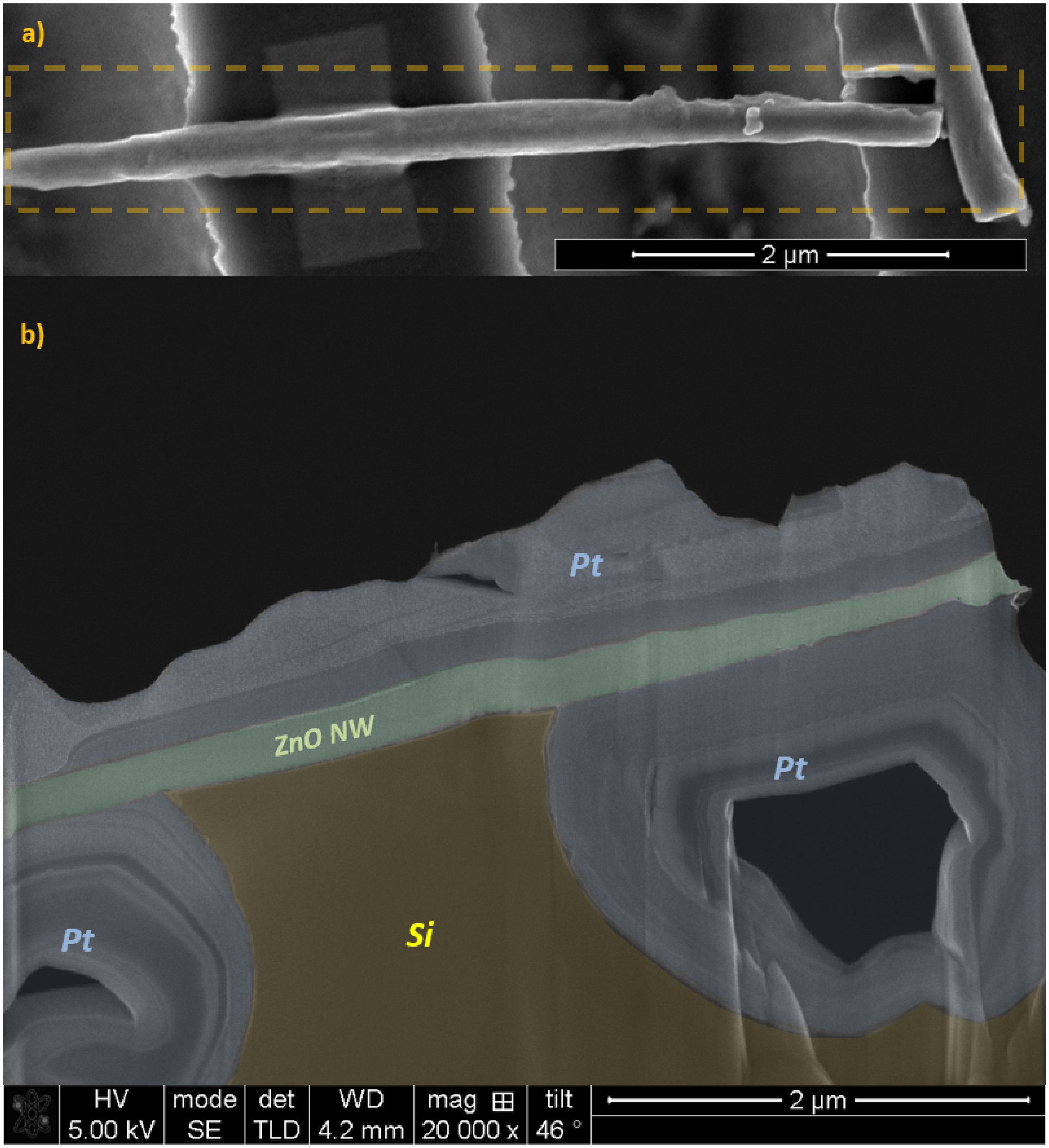

Figure 1.3: Scanning electron microscopy image a) of the plastically deformed ZnO NW III observed from the top and b) of a TEM lamella of the same NW prepared by FIB mciromachining. Platinum was deposited prior to the milling process to avoid any damaging of the NW.

Fig. 1.3(a) presents a *post-mortem* SEM image of the plastically deformed ZnO NW III. A transmission electron microscopy lamella of the abovementioned ZnO NW III prepared by focused ion beam (FIB) micromachining is displayed in Fig. 1.3(b) showing the left-hand Si support and the

suspended NW part on the right-hand side. A platinum layer was priorly deposited on top of the NW and its surrounding to protect it against the Ga+ ions during FIB milling.

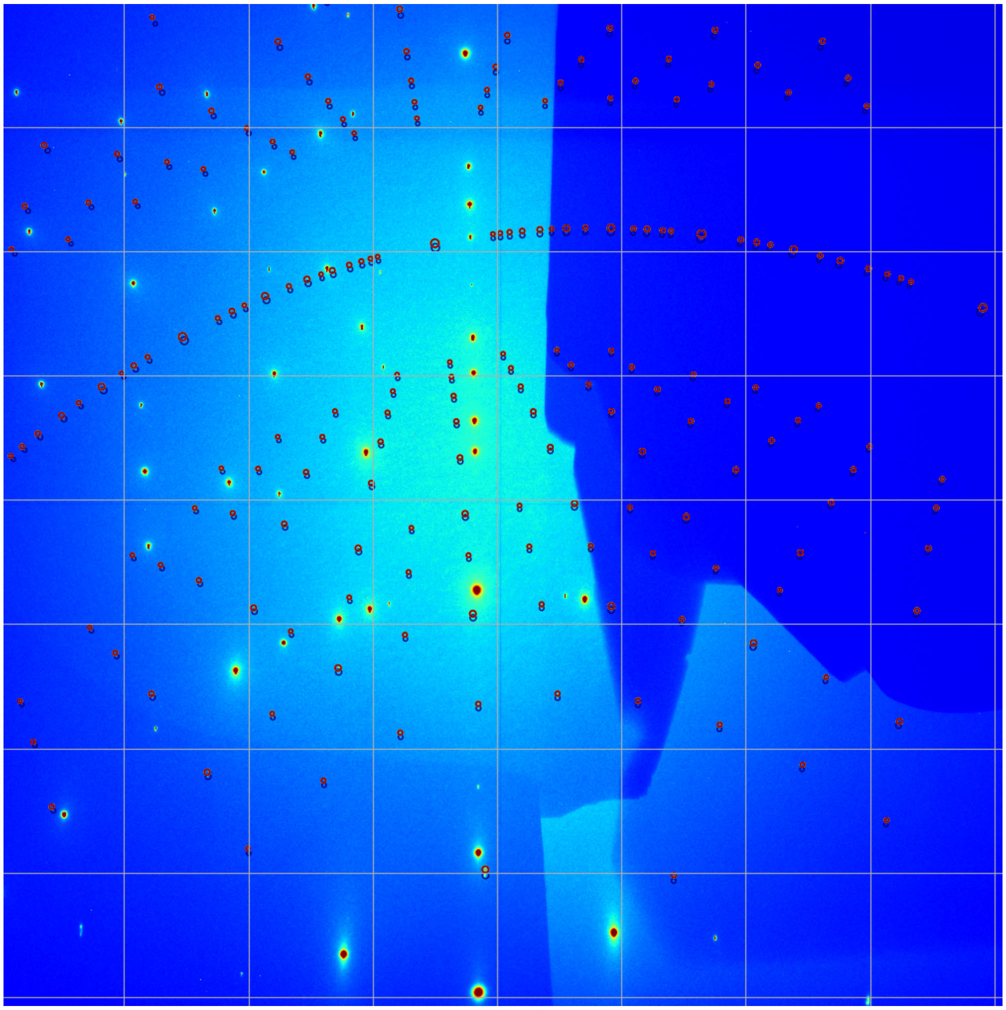

Figure 1.4: Complete Laue microdiffraction pattern of the ZnO NW III overlaid with the simulated diffraction pattern of a ZnO crystal with the exact same orientation and a second ZnO crystal rotated by 0.20° around the c-axis.

A complete Laue microdiffraction pattern of the ZnO NW III overlaid with the simulated diffraction pattern of a ZnO crystal with the exact same orientation (represented by blue circles) and a second ZnO crystal rotated by 0.20° around the c-axis (represented by red circles) is displayed in 1.4. The simulated diffraction patterns superimpose with the experimental Laue spots evidencing that the subgrain formation induced the rotation of the ZnO NW crystal around the [0001] axis by 0.20°.

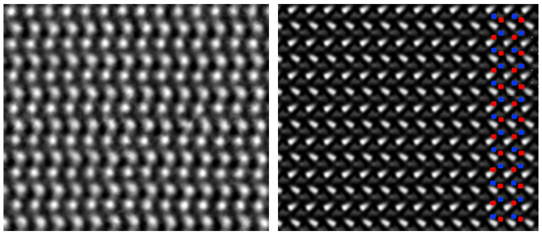

Figure 1.5: Experimental (left) and simulated (right) HRTEM images of the wurtzite structure observed along the <$2\bar{1}\bar{1}0$> crystallographic axis. The thickness of the sample is estimated to 17.2 nm in this region (C1: -23nm, C3: -7μm). The atomic positions of Zn (blue) and O (red) atoms are superimposed to the right-part of the simulated image.

A HRTEM image of the ZnO structure of the bent ZnO NW III is presented in 1.5 on the left. For comparison, a simulated image with slightly different contrast is presented in 1.5 on the right. The simulated image helps to estimate the thickness of the probed region which amounts here to about 17.2 nm.
